# Determining the dynamics of influenza transmission by age

**DOI:** 10.1186/1742-7622-11-4

**Published:** 2014-03-21

**Authors:** Laura F White, Brett Archer, Marcello Pagano

**Affiliations:** 1 Department of Biostatistics, Boston University School of Public Health, 801 Massachusetts Ave, Boston, MA 02118, USA; 2 National Institute of Communicable Diseases, National Health Laboratory Service, Johannesburg, South Africa; 3 Department of Biostatistics, Harvard School of Public Health, 655 Huntington Ave, Boston, MA 02115, USA

**Keywords:** Pandemic influenza H1N1, Reproductive number, Infectious disease

## Abstract

**Background:**

It is widely accepted that influenza transmission dynamics vary by age; however methods to quantify the reproductive number by age group are limited. We introduce a simple method to estimate the reproductive number by modifying the method originally proposed by Wallinga and Teunis and using existing information on contact patterns between age groups. We additionally perform a sensitivity analysis to determine the potential impact of differential healthcare seeking patterns by age. We illustrate this method using data from the 2009 H1N1 Influenza pandemic in Gauteng Province, South Africa.

**Results:**

Our results are consistent with others in showing decreased transmission with age. We show that results can change markedly when we make the account for differential healthcare seeking behaviors by age.

**Conclusions:**

We show substantial heterogeneity in transmission by age group during the Influenza A H1N1 pandemic in South Africa. This information can greatly assist in targeting interventions and implementing social distancing measures.

## Background

The importance of the dynamics of influenza transmission between age groups is well-appreciated [1-6]. Several studies have assessed the non-uniformity of the impact of influenza, particularly pandemic influenza, on different age groups [1-4,7-9]. The overarching interest in these studies is to gather information in order to influence policy to best determine a strategy to impact on the spread of outbreaks. Which age groups carry the greatest disease burden and which groups are responsible for the greatest amount of disease transmission is an important component of this information.

One key aspect of this work is to estimate the extent to which people in different age groups interact with one another and to what degree they are in contact. This information can then be used as a surrogate for transmission probabilities between age groups [10-12]. Several studies have generated matrices with estimated numbers of contacts between various age groups [5,12,13]. Additionally, social network models have been used to estimate these contact patterns [7]. A common finding amongst these studies is that children tend to mix mostly with each other, and to a lesser extent with their parents, while adults mix with individuals from a larger range of ages [5,7]. These matrices have subsequently been used in modeling exercises to better understand the dynamics of disease spread by age.

When determining which groups to target for interventions in an outbreak, one strategy is to target those who potentially carry the greatest burden of disease [14], which has typically been found to be children [1,5,7,8,13]. For instance, Bansel *et al.*[4] consider data from influenza pandemics over the past century and show that the burden of disease is highest amongst children during a pandemic and then shifts to adults the following season.

To better understand the utility of targeting the groups with the greatest burden of disease, it is also important to determine when different age groups tend to have their peak incidence of cases. This can also be seen as a surrogate measure for the age group that is driving an outbreak [15-17]. Most recently Schanzer et al. [16] used 10 years of Canadian surveillance data of laboratory confirmed cases of influenza and found that during seasonal influenza the 10–19 and 20–29 year-old age groups tended to peak one week earlier than other groups. During the pandemic in 2009, the peak came earliest for only the 10–19 year old age group. This is inconsistent with the findings of Brownstein et al. [15] who found that children aged 3–4 were consistently the first to peak.

A different tactic for determining which groups should be the target of interventions is to select those groups most responsible for transmission [18]. Studies examining disease transmission by age have consistently shown that children have higher estimated values of the reproductive number than adults. Recent work has focused on the dynamics during the 2009 Influenza pandemic. During the initial phase of the pandemic in Japan, Nishiura et al. [19] report that children were transmitting illness at higher rates than adults. Glass et al. [2] used Japanese data and a novel method to estimate the reproductive number, R_0_, for adults and children that assume particular forms for a next-generation matrix and estimate the parameters of this matrix, leading to outbreak specific estimates of R_0_. They estimate R_0_ to be between 2.8 and 3.6 for children and between 0.2 and 0.7 for adults, depending on the assumptions made. In a later study, Glass et al. [3] used serosurvey data and estimate R_0_ from the final size of the epidemic to be 1.6 for kids and less than 1 for adults. Wallinga et al. [18] have similarly shown that the rate in change of the reproductive number for a particular group is related to the incidence of infection and force of infection and suggest allocating resources based on examining these two quantities.

In the present study, we present a new approach to estimating the reproductive number by age group by modifying a method initially proposed by Wallinga and Tuenis [20]. We study age dynamics in South Africa during the 2009 Influenza H1N1 pandemic and illustrate the importance of an appropriately estimated measure of the transmission dynamics on final estimates. Finally, we discuss our results and their implications for future studies on how to respond during an emerging outbreak.

## Methods

### Data

We use de-identified data previously reported in [21] that includes a line list of the 12,543 confirmed cases reported in South Africa during that outbreak. Included in the data are the ages of the individuals, the provinces where the specimens were collected, the sex of the individuals, the dates of onset of symptoms, and the dates of the reporting of specimens. The information on the date of symptom onset was reported for 758 cases (6%). We use multiple imputation techniques to create 500 different datasets with the missing onset times imputed, as predicted by the province and an indicator of whether the specimen was collected on a weekday or weekend, using Poisson regression [22]. We report the averages and ranges over the 500 imputed datasets. Contact tracing information was collected on 100 initial cases, to provide an estimate of the serial interval, as has been previously reported [21]. We only use data from Gauteng province (n = 5579, 44% of cases) to avoid confounding the results with potential spatial variation in transmission. Gauteng province is the most populous, yet smallest geographically, of the nine provinces in South Africa, with over 10 million inhabitants, predominantly in the cities of Johannesburg and Pretoria.

### Statistical methods

Wallinga and Teunis [20] (denoted WT method hereafter) proposed a method for the estimation of the effective reproductive number by making use of the epidemic curve, N = {N_1_,…, N_T_}, where N_t_ is the number of cases at time point t, and an estimate of the serial interval, p_1_,…, p_k_, where p_i_ describes the probability of a serial interval of length i and the maximum serial interval length is k. We review this method in Appendix 1. The estimator they obtain for the effective reproductive number for individual j on day t’ is

Rtj'=Σs=t'+1minT,t'+kΣi=1nsqsi,tj'=Σs=t'+1minT,t'+knsqs,tj',

where n_s_ denotes the number with symptom onset on day s and q_s,t_ denotes the relative probability that case s was infected by case t.

### Age transmission data

We propose the use of additional structure in this method to describe the probability of an infection event occurring between two cases that incorporates information on their ages by modifying the probability of transmission to be:

Ptj'→ti=pti−tj'wai'aj,

where a_j_ is the age group of individual j and wai'aj is a measure of the likelihood of transmission between individuals in age group a_i_ and a_j_. The matrix W = {$w_{a_{i}a_{j}}$} does not necessarily have to be symmetric.

This method requires information on the likelihood of infectious contact between different age groups, or the $w_{a_{i}a_{j}}$. Increasingly studies are being conducted to obtain such information by assuming that transmission is directly related to contact patterns. We use the results of two such studies:

The first is a study of 571 randomly selected individuals in a South Africa township performed in 2010 and reported by Johnstone-Robertson et al. [12]. The authors report two matrices with age specific contact patterns in five year intervals up to a 45+ category. The first matrix only considers contacts that involve all close contacts while the second includes information on only those contacts that involve physical touch.

The second set of matrices we use comes from the European based PolyMod study of Mossong et al. [5]. This study includes information on 97,904 contacts amongst 7,290 participants from eight countries in Europe: Belgium, Finland, Great Britain, Germany, Italy, Luxembourg, the Netherlands, and Poland. Contact matrices describe all close contacts, and then separately, close contacts that involve physical touch. The matrices report age-specific values for five year age groups up to 70+. We modify these matrices to match those presented by Johnstone-Robertson et al. [12] and to match the demographics of South Africa’s young population by averaging all values above 45 years of age to create a single 45+ age category. In our results we focus on those obtained using the contact matrices from South Africa, as these matrices would seem more appropriate for the data at hand. We report results from the PolyMod matrices as a sensitivity analysis.

We estimate R_t_ and R_0_ using the 18 matrices described above with the imputed epidemic data from South Africa, and report age specific estimates of these quantities, as well as aggregate estimates across age groups. The reproductive numbers for each age group represent the expected number of infections generated across the population by an individual in that particular age group.

In the Appendices, we further report the results of two sensitivity analyses: First we test the sensitivity of the results to potential errors in the reporting dates by selecting a single imputed dataset and randomly jittering the onset dates of 10% of the individuals, within 30 days of their observed (or imputed) onset date (Appendix 2). We create 50 such datasets and repeat all analyses on these datasets and compare these results to those obtained without jittering the data. The second sensitivity analysis tests the impact of differential healthcare seeking behaviors by age. We smooth the distribution of the proportion of cases that were hospitalized by age group to serve as a surrogate distribution of healthcare seeking behavior and/or reporting patterns by age. This distribution is U-shaped, indicating that the very young and very old are more likely to seek medical care, a finding that has been reported elsewhere [23]. We attach various weights to this distribution and augment 25 of our imputed datasets according to this distribution. We reanalyze this augmented data to determine the potential impact of differential case reporting by age group on the results (Appendix 3).

## Results

**Figure 1:**
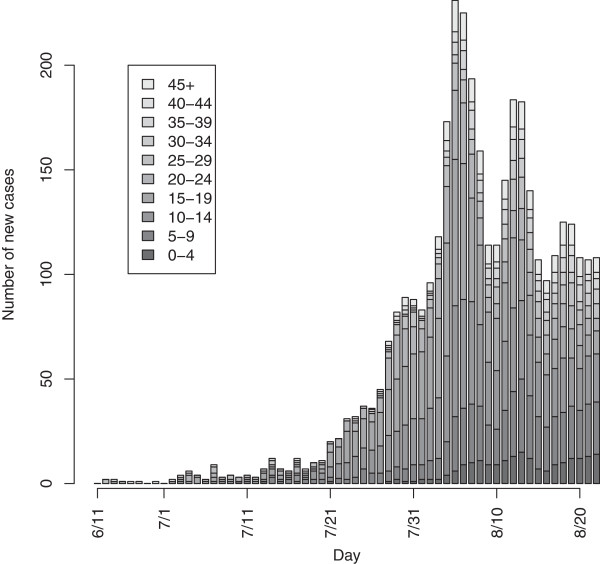
Epidemic curves by age group.

Figure 1 provides the epidemic curves across all age groups. Here, school age children and young adults tend to have the greatest number of cases initially in the outbreak.

**Figure 2:**
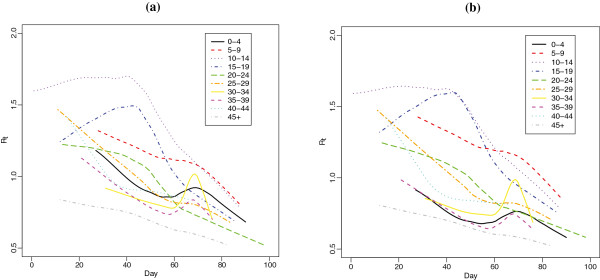
**Smoothed estimates of R**_**t **_**for the transmission matrix based on (a) all contacts involving physical contact and (b) all close contacts using the South African contact matrices.**

Figure 2 shows the estimated effective reproductive numbers for the two Johnstone-Roberston et al. matrices. The ${\hat{R}}_{t}$ estimates are similar regardless of the type of contact matrix assumed (close contact versus those involving physical contact). Overall, ${\hat{R}}_{t}$ is much higher for those in the 15–19 and 20–24 year old groups throughout much of the epidemic, with the 10–14 and 25–29 year old age groups rapidly achieving high values, as well. Those over 45 initially have fairly high estimates of ${\hat{R}}_{t}$ but these taper off quickly. Estimates of ${\hat{R}}_{t}$ are not obtainable for those between 5 and 9 and those less than 5 until the outbreak is well under way, due to the paucity of observed cases for those age groups early on in the epidemic.

**Figure 3:**
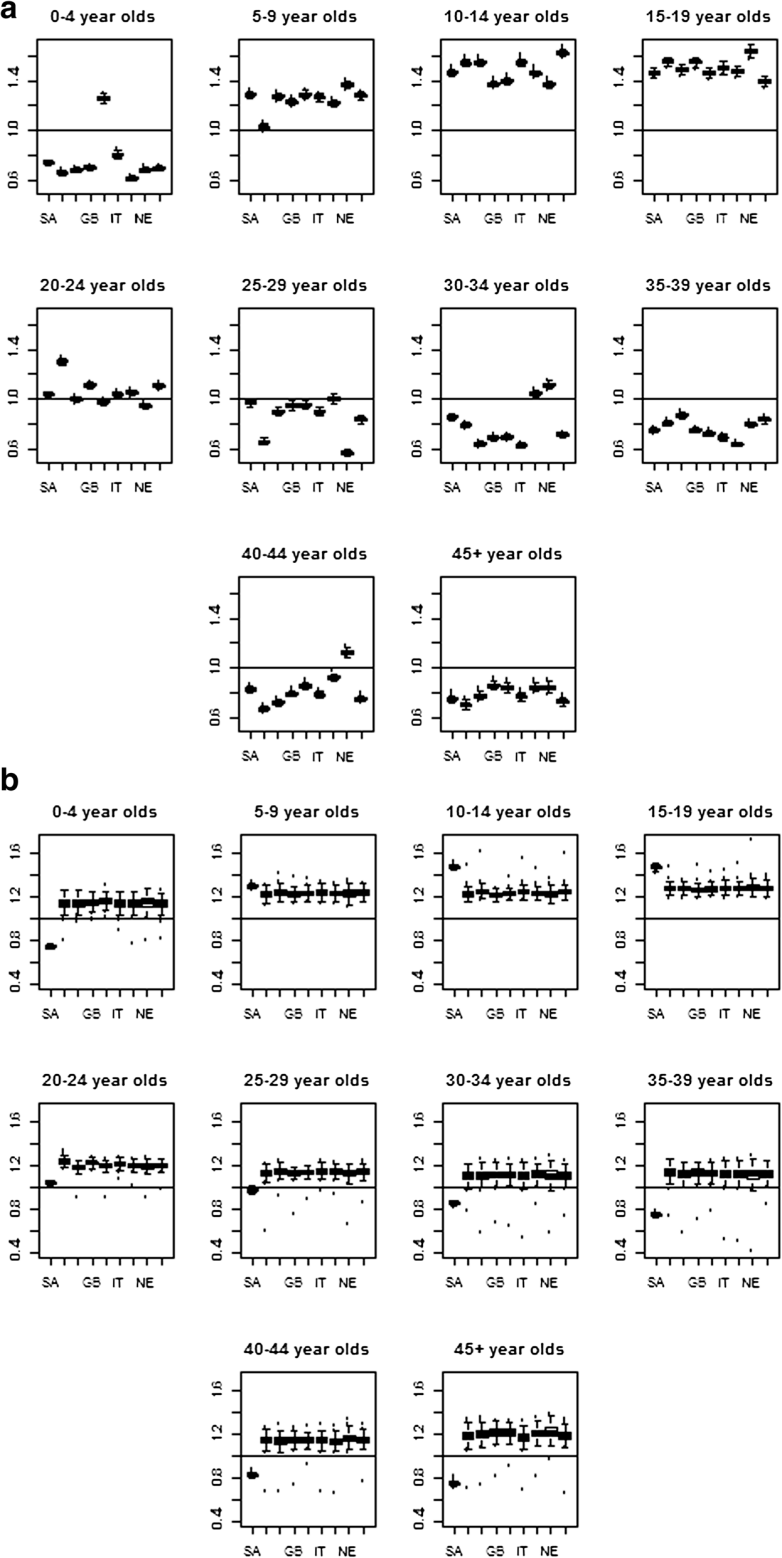
Estimated R0 by age group for each of the contact trace matrices from the nine countries in the PolyMod study using (a) all contacts, and (b) physical contacts.

We obtain estimates of R_0_, the basic reproductive number, by averaging the estimates of R_t_ during the epidemic period. In reality this can be viewed as a pseudo-R_0_ given the prior immunity to this strain of influenza. We will refer to it as R_0_ throughout the text. We assume that the epidemic period corresponds to the point at which transmission was sustained in Gauteng Province until the overall number of cases peaked. This corresponds to the period between 22 June 2009 and 21 August 2009. Figure 3 and Table 1 show the estimates of R_0_ across age groups along with the number of individuals in each age group who were reported infected throughout the epidemic. Regardless of the choice of matrix, supercritical values of R_0_ are obtained for those between the age of 5 and 24, with the highest values being observed for those in the 10–14 age-group (R_0_ = 1.53 for close contacts).

**Table 1:** **Estimates of R**_**0 **_**obtained by using the transmission matrices based on South African contact patterns**

**Age group**	**N (%)**	${\hat{R}}_{0}$ **, close contacts**	${\hat{R}}_{0}$ **, all physical contacts**
0-4	484 (8.73)	0.94 (0.91-0.97)	0.74 (0.72-0.76)
5-9	927 (16.72)	1.20 (1.17-1.24)	1.29 (1.25-1.33)
10-14	1150 (20.75)	1.53 (1.49-1.58)	1.47 (1.44-1.51)
15-19	1026 (18.52)	1.36 (1.32-1.40)	1.47 (1.42-1.50)
20-24	556 (10.03)	1.06 (1.03-1.09)	1.03 (1.01-1.06)
25-29	389 (7.02)	0.98 (0.94-1.01)	0.97 (0.94-1.01)
30-34	229 (4.13)	0.92 (0.88-0.94)	0.86 (0.82-0.88)
35-39	246 (4.44)	0.85 (0.82-0.88)	0.75 (0.82-0.78)
40-44	171 (3.09)	0.86 (0.83-0.90)	0.83 (0.80-0.87)
45+	363 (6.55)	0.79 (0.75-0.85)	0.75 (0.71-0.81)

**Figure 4:**
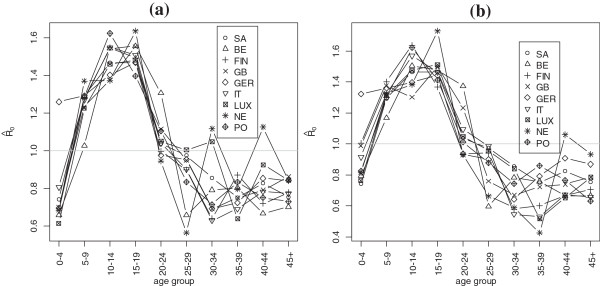
**Estimates of R**_**0 **_**by age groups using contact matrices from South Africa and separately each of the eight countries in the Mossong et al. study. (a)** results for all close contacts and **(b)** for contacts involving physical touch.

We contrast these estimates with those obtained using contact matrices from Europe [5]. Figures 3a and 3b shows the estimates of R_0_ across the 10 age groups obtained when using contact patterns from South Africa and the eight European countries in the PolyMod study for all close contacts (Figure 3a) and all contacts involving physical touch (Figure 3b). There are few notable differences between the estimates. In Figure 4, the mean estimates of R_0_ are shown for each age group. We observe a similar overall trend for the estimate of R_0_ across the age groups.

Finally we provide the overall estimate of R_0_ collapsed over all age groups (Table 2). For comparison purposes, we first estimate R_0_ by using a traditional analysis that assumes homogenous mixing among the age groups (${\hat{R}}_{0}$=1.28, range: 1.26-1.31). This is similar to that obtained for all the other contact matrices considered. Additionally there are virtually no differences observed between results from the two contact matrices.

**Table 2:** **Overall estimate of R**_**0 **_**for the different methods used**

**Method**	**Overall** ${\hat{R}}_{0}$	
Homogenous mixing	1.28 (1.26-1.31)	
Contact matrix used	All close contacts	Contacts with physical touch
South Africa	1.27 (1.25-1.31)	1.27 (1.25-1.31)
Belgium	1.26 (1.24-1.31)	1.27 (1.24-1.31)
Finland	1.27 (1.25-1.32)	1.27 (1.25-1.32)
Great Britain	1.27 (1.25-1.32)	1.27 (1.25-1.31)
Germany	1.27 (1.25-1.32)	1.27 (1.25-1.32)
Italy	1.27 (1.25-1.31)	1.27 (1.24-1.31)
Luxembourg	1.27 (1.25-1.32)	1.27 (1.25-1.32)
Netherlands	1.27 (1.25-1.32)	1.27 (1.25-1.32)
Poland	1.27 (1.25-1.31)	1.27 (1.24-1.31)

Ranges give the values obtained across the 500 imputations.

Our first sensitivity analysis, which jitters the onset dates of a subset of the population, (Additional file 1: Table S1) provides results that are consistent with the results presented. Not surprisingly, the impact of reassigning onset dates to a portion of the dataset has the impact of flattening the epidemic curve and thus lowering the estimates of R_0_. However the results remain consistent and, coupled with the imputation variability reported, provide insight on the overall variability of the estimates reported.

In our second sensitivity analysis (Additional file 2: Table S2 and Additional file 3: Figure S1), examining the potential impact of differential reporting by age, we note a dramatic impact on the results. As we assume a greater underreporting of cases among those who are middle aged, we estimate the bulk of transmission being attributable to those who are older and less transmission being attributed to the very young, a finding contrary to the original results we present.

## Discussion

We present a novel approach to estimating the effective and basic reproductive number by age group, and have applied this method to data from the 2009 Influenza H1N1pdm in Gauteng Province in South Africa. This method requires some estimate of contact patterns between age groups. We show results for 18 different possible contact matrices and the impact that these matrices have on the estimates. Additionally, as with the original method proposed by Wallinga and Teunis [20], it is necessary to have an estimate of the serial interval and we use an estimate obtained from contact trace data in South Africa.

As has been previously noted, that the burden of disease appears to be greatest amongst the young [21], a finding consistent with other studies [1,4,7]. These data argue that aiming interventions at youth would target the group that carries the largest burden and should have the best chance of success in limiting transmission. This finding is consistent with the strategy proposed by Wallinga et al. [18] and provides further information in the form of actual estimates of the reproductive number.

Our results also illustrate the importance of accounting for the age structure when estimating reproduction numbers. Though the overall estimates of R_0_ are unaffected by the incorporation of this information, we obtain much richer information with the ability to obtain age-specific estimates of the reproductive number. This analysis provides greater insight into the dynamics of disease transmission and informs intervention strategies.

The results obtained using information on transmission dynamics from the study based in South Africa [12] as well as that of the PolyMod study [5], appear to corroborate previous results for influenza pandemics [2,3,19] which seem to imply that school aged children are responsible for the bulk of disease transmission. Specifically we estimate that ${\hat{R}}_{0}$ is highest for 10–14 year olds when using South African contact trace matrices (close contacts: ${\hat{R}}_{0}$ = 1.53, range, 1.49-1.58; physical touch contacts: ${\hat{R}}_{0}$ = 1.47, range, 1.44-1.51). These results are similar to the results from other studies [2,19] and those obtained using the European based contact matrices.

Interestingly, it does not appear to make a substantial difference which contact pattern matrix we use in our analysis. One would assume that the matrices obtained in South Africa would be most relevant to the outbreak data we are analyzing and indeed, we have chosen to present the majority of our results using these matrices. We note, however, that when we use contact patterns from other European countries, where the demographics, climate, healthcare system, government, overall health, etc. are different from that of South Africa, there are only minor changes in the results. Indeed, the contact patterns observed in the Polymod study and the South African contact study are not substantially different, however they are not identical. This appears to argue that using some form of adjustment is superior to assuming homogenous mixing, but the method we propose is not overly sensitive to the form the adjustments take [24]. This result is similar to that of Glass et al. [2] who experimented with four forms of next generation matrices to estimate the reproductive number for adults and children separately. They found that the estimates of the reproductive numbers were not overly sensitive to the matrix forms that they assumed.

However, one should still take care in the assumptions used when implementing this method, or others like it. Our study is only one instance and it is not clear that the results we obtain would replicate in other settings. For instance, if one were to always use the Polymod study information for studies throughout the world, there is still the potential for errors if contact patterns do differ dramatically from those observed in Europe. While it is impossible to know with certainty if this is the case without detailed contact pattern information for the area of study, one can, at the least conduct a sensitivity analysis to determine the potential impact of the contact matrix on the analysis. Additionally we implicitly assume that contact patterns are directly related to transmission probabilities, an assumption that has yet to be rigorously tested. There is also work to show that contact patterns can change considerably during illness [25,26]. Our work relies on the contact patterns of healthy individuals. While we suspect, based on our sensitivity analyses, that this will not have a substantial impact on our results, this is important to note.

It is important to note the caveats and limitations of this study. Our results would be impacted if reporting was inconsistent throughout the outbreak among the age groups. For instance if reporting was very good among one age group initially but declined in quality as the outbreak progressed, we can expect that our results would be biased [27]. In general the default assumption is that the pyramid of disease reporting described in [28,29] is the same for all age groups. Our second sensitivity analysis (Appendix 3) explores the impact of this assumption and shows that if reporting or healthcare seeking behavior is much lower among middle aged groups than the very old and very young, our results will change dramatically. In the extreme case, we see that transmission is mostly attributable to those who are at least 30 years old and that the very young are unable to sustain transmission. While this result is contrary to what has been reported in the scientific literature to date, the potential for reporting inconsistencies that we explore are not unlikely, and have not been recognized and corrected for in other analyses that we are aware of. Brooks-Pollock et al. [23] report results from a survey conducted during the 2009 H1N1 Influenza pandemic in the UK that showed that the very young and very old were more likely to seek healthcare when ill. The impact of correcting surveillance data to accommodate this phenomenon was to shift the burden of illness from the very young to the middle-aged. Further investigation into potential reporting inconsistencies is important to better understand infectious disease dynamics by age similar to what was previously done but not incorporating age [27]. Another reporting issue arises from silent infections, or those who carry infection and have the potential to transmit it, but are asymptomatic. We did not investigate the impact of these individuals, though the issues are similar to those we have just described.

Additional reporting inconsistencies are possible spatially or across other socio-economic factors. Our analysis was only performed on data from Gauteng province, the most urban province in South Africa. It is possible that reporting would not be as dramatically variable as it would be if we were to make use of data from the entire country. It is also important to note that we chose to limit our analysis to Gauteng province so as to limit the impact of spatial effects and make the assumption of homogenous mixing more reasonable. This could limit generalizability.

We have also assumed that the contact matrices we use are correct and do not allow for any uncertainty in their estimation. These results might be improved upon and made more realistic by allowing for greater stochastic effects and/or flexibility in the transmission matrix. Ideally we would estimate these parameters in our study, but we do not have sufficient data to do so in the present framework. Glass et al. [2] have shown how to do this for a matrix with adults and children, but are limited to two by two matrices that presume a pre-specified structure and are unable to consider a larger number of age groups, thus limiting their ability to gain a more thorough and detailed understanding of transmission.

## Conclusions

We have applied a novel method to estimate transmission patterns between individuals from different age groups during the 2009 Influenza H1N1pdm in South Africa. We show that assumptions regarding the assumed contact patterns between age groups do not substantially impact the conclusions one draws from the data analyses in our study. Our results are consistent with other studies that show children are much more likely to become ill and transmit disease than adults during a pandemic, if the completeness of the data reported is independent of the age of the patients. These methods can be used to estimate heterogeneity in transmission parameters in real time by using the modification proposed by Cauchemez et al. [30] and thus inform the use of targeted interventions by age group.

## Appendix 1

### Wallinga and Teunis method

Wallinga and Teunis (20) (denoted WT method hereafter) proposed a method for the estimation of the effective reproductive number by making use of the epidemic curve, **N** = {N_1_,…, N_T_}, where N_t_ is the number of cases at time point t, and an estimate of the serial interval, p_1_,…, p_k_, where p_i_ describes the probability of a serial interval of length i and the maximum serial interval length is k. For ease of presentation, we assume that the time step is a day. Individuals are placed in a network temporally by symptom onset date and the probability of transmission occurring between two individuals in the network is determined by the serial interval. The calculation of R_t_ occurs in three steps. In what follows, we let t_i_ denote the i^th^ individual with symptom onset on day t, where i = 1,…,N_t_.

1. For the i^th^ individual with symptom onset on day t, calculate the probabilities of infection by all those with symptom onset on prior days t^2^ (t2 < t.) These probabilities equal the serial interval probability for the distance in time between the potential infector, t_j_2 , and infectee, t_i_, P (t_j_' →t_i_) = $p_{t_{i}-t_{j}^{'}}$.

2. Calculate the relative probability that the case t_i_ was infected by the j^th^ case on day P (t_j_' →t_i_), denoted by qti,tj',

qti,tj'=Ptj'→ti∑s=1mink,ti−1∑l=1nsPsl→ti=Ptj'→ti∑s=1mink,ti−1nsPs→ti.

3. Calculate the reproductive number. For the j^th^ case on day t', say t_j_', the reproductive number is calculated as the sum of the expected values of a Bernoulli random variable. The Bernoulli random variable describes the event that t_j_' infected another individual on day s, s > t', say s_i_, and has probability q_s,t'_. Then the effective reproductive number for individual t_j_′is

Rtj'=Σs=t'+1minT,t'+kΣi=1nsqsi,tj'=Σs=t'+1minT,t'+knsqs,tj',

where n_s_ denotes the number with symptom onset on day s.

## Appendix 2

### Sensitivity analysis: Impact of errors in reporting dates

In this analysis, we choose a single imputed dataset and randomly jitter the onset dates of 10% of the sample within 30 days of their observed (or imputed) onset date. We create 50 such datasets and repeat all analyses on these datasets and compare them to the results on the non-jittered dataset.

## Appendix 3

### Sensitivity analysis: Impact of differential reporting by age

We assume that the reporting distribution by age follows a U-shaped distribution, implying that the very young and very old are most likely to seek healthcare and have their cases reported. To obtain a distribution that follows this shape, we use the distribution of hospitalized cases by age in our data, rescale it so that the highest proportion is one, and smooth the distribution using a loess smoother (Additional file 3: Figure S1).

We use 25 of our imputed datasets and augment each dataset using the distribution f (x), where f (x) is a function of the original age distribution observed in the data, g (x), and the reporting distribution shown in Additional file 3: Figure S1, h (x), as follows:

$$f\left(x\right)=\mathrm{\lambda g}\left(x\right)+\left(1-\lambda \right)h\left(x\right).$$

Here *λ* ranges between 0 and 1. We run analyses for *λ* = 0.0, 0.25, 0.50, 0.75 and 1.0 (corresponding to the original analysis). Results for all 25 datasets are shown in Additional file 2: Table S2.

### Competing interests

The authors report no conflicts of interest.

### Authors’ contributions

LFW and MP conceived the project and developed the methods. LFW performed the analyses. BA provided the data and insight on dynamics in South Africa. LFW drafted the manuscript. All authors read and approved the final manuscript.

## Supplementary Material

Additional file 1: Table S1 Results for the sensitivity analysis using the South African based age contact information. Result presented is the estimate obtained from the original dataset and the values in the parentheses represent the range of values obtained over the 50 datasets generated for the sensitivity analysis. Click here for file

Additional file 2: Table S2 Estimates of R_0_ using 25 of the 500 imputations described in the original text. Results shown are the mean and range of estimates across the 25 imputed datasets. λ=1.00 corresponds to the results from the original analysis. Click here for file

Additional file 3: Figure S1 Smoothed distribution to reflect potential rates of healthcare seeking behavior and/or case-reporting by age. Click here for file
