# Supplementary material for: Determining the dynamics of influenza transmission by age
Source: Emerg Themes Epidemiol. 2014 Mar 21;11:4. doi: 10.1186/1742-7622-11-4 (PMC3997935; doi:10.1186/1742-7622-11-4)
Supplement: Additional file 1: Table S1 — Results for the sensitivity analysis using the South African based age contact information. Result presented is the estimate obtained from the original dataset and the values in the parentheses represent the range of values obtained over the 50 datasets generated for the sensitivity analysis. [file 1742-7622-11-4-S1.doc]

**Table S1**. Results for the sensitivity analysis using the South African based age contact information. Result presented is the estimate obtained from the original dataset and the values in the parentheses represent the range of values obtained over the 50 datasets generated for the sensitivity analysis.

| Age group | , close contacts | , all physical contacts |
| --- | --- | --- |
| Overall | 1.26 (1.06-1.26) | 1.26 (1.06-1.26) |
| 0-4 | 0.94 (0.72-0.95) | 0.74 (0.56-0.75) |
| 5-9 | 1.21 (0.99-1.21) | 1.30 (1.06-1.31) |
| 10-14 | 1.50 (1.24-1.52) | 1.44 (1.20-1.46) |
| 15-19 | 1.35 (1.15-1.35) | 1.45 (1.23-1.45) |
| 20-24 | 1.03 (0.92-1.10) | 1.01 (0.90-1.07) |
| 25-29 | 0.96 (0.78-0.96) | 0.95 (0.77-0.96) |
| 30-34 | 0.92 (0.80-1.00) | 0.86 (0.75-0.93) |
| 35-39 | 0.84 (0.68-0.86) | 0.74 (0.59-0.75) |
| 40-44 | 0.87 (0.69-0.87) | 0.84 (0.66-0.84) |
| 45+ | 0.77 (0.62-0.77) | 0.73 (0.59-0.74) |
