# Supplementary material for: Determining the dynamics of influenza transmission by age
Source: Emerg Themes Epidemiol. 2014 Mar 21;11:4. doi: 10.1186/1742-7622-11-4 (PMC3997935; doi:10.1186/1742-7622-11-4)
Supplement: Additional file 2: Table S2 — Estimates of R0 using 25 of the 500 imputations described in the original text. Results shown are the mean and range of estimates across the 25 imputed datasets. λ=1.00 corresponds to the results from the original analysis. [file 1742-7622-11-4-S2.doc]

**Table S2**. Estimates of R_0_ using 25 of the 500 imputations described in the original text. Results shown are the mean and range of estimates across the 25 imputed datasets.
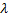
=1.00 corresponds to the results from the original analysis.

| Age group | 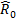, (range across 25 imputations used) | | | | |
| --- | --- | --- | --- | --- | --- |
|  | 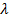=0.0 | 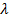=0.25 | 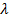=0.50 | 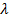=0.75 | 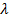=1.00 |
| Overall | 0.52  (0.51-0.53) | 0.71  (0.69-0.73) | 0.89  (0.88-0.92) | 1.1  (1.10-1.15) | 1.38  (1.37-1.40) |
| 0-4 | 1.17  (1.14-1.20) | 0.87  (0.85-0.89) | 0.75  (0.74-0.77) | 0.69  (0.67-0.70) | 0.74  (0.73-0.76) |
| 5-9 | 0.49  (0.48-0.50) | 0.53  (0.52-0.54) | 0.64  (0.61-0.66) | 0.71  (0.69-0.73) | 1.29  (1.27-1.31) |
| 10-14 | 0.45  (0.44-0.45) | 0.60  (0.59-0.61) | 0.77  (0.76-0.78) | 0.99  (0.95-1.01) | 1.47  (1.44-1.51) |
| 15-19 | 0.73  (0.72-0.75) | 0.85  (0.83-0.86) | 1.04  (1.00-1.07) | 1.28  (1.23-1.32) | 1.46  (1.42-1.48) |
| 20-24 | 0.79  (0.77-0.80) | 0.88  (0.86-0.89) | 1.00  (0.99-1.03) | 1.26  (1.23-1.29) | 1.04  (1.01-1.06) |
| 25-29 | 1.28  (1.24-1.31) | 1.22  (1.17-1.29) | 1.13  (1.07-1.19) | 1.16  (1.10-1.20) | 0.97  (0.95-1.00) |
| 30-34 | 1.77  (1.80-1.82) | 1.37  (1.32-1.40) | 1.21  (1.18-1.25) | 1.12  (1.07-1.15) | 0.86  (0.83-0.88) |
| 35-39 | 1.61  (1.58-1.66) | 1.19  (1.15-1.22) | 1.04  (1.02-1.06) | 0.95  (0.93-0.97) | 0.75  (0.73-0.77) |
| 40-44 | 1.56  (1.52-1.59) | 1.16  (1.14-1.17) | 1.00  (0.97-1.01) | 0.90  (0.88-0.92) | 0.83  (0.80-0.84) |
| 45+ | 1.31  (1.28-1.35) | 1.01  (0.98-1.05) | 0.89  (0.86-0.93) | 0.82  (0.79-0.86) | 0.76  (0.74-0.80) |
